# Partner Care Arrangements and Older Europeans' Well-Being: Variations by Gender and Context

**DOI:** 10.1093/geroni/igab046.1520

**Published:** 2021-12-17

**Authors:** Ginevra Floridi, Nekehia Quashie, Karen Glaser, Martina Brandt

**Affiliations:** 1 Nuffield College, University of Oxford, Oxford, England, United Kingdom; 2 Technische Universitat Dortmund, Dortmund, Nordrhein-Westfalen, Germany; 3 King's College London, London, England, United Kingdom

## Abstract

Across Europe, partners are often primary caregivers to older adults with care needs. Yet, a variety of partner care arrangements may arise. Little is known about the interrelations between partners’ care arrangements, (potential) caregivers’ gender, and the context in which care is embedded. We use 2015 SHARE data from 17 countries on 3,465 couples aged 50+ where one partner receives care. We examine how life satisfaction and depressive symptoms of (potential) caregivers vary across five care arrangements: solo-care; shared formal; shared informal; outsourced formal; and outsourced informal. We explore heterogeneity by gender and across four contexts: Northern, Western, Southern, and Eastern Europe. Outsourcing partners’ care to formal or informal providers is linked with higher well-being among Northern and Western European women, but with lower well-being among women in Southern Europe, where traditional female caregiving responsibilities are stronger. Among men, outsourcing partner care is linked to higher well-being regardless of context.

